# Propolis induces cardiac metabolism changes in 6-hydroxydopamine animal model: A dietary intervention as a potential cardioprotective approach in Parkinson’s disease

**DOI:** 10.3389/fphar.2022.1013703

**Published:** 2022-10-13

**Authors:** Valeria C. Goncalves, Victor Silva da Fonsêca, Daniele de Paula Faria, Mario Augusto Izidoro, Andresa Aparecida Berretta, Antônio-Carlos G. de Almeida, Fernando Luiz Affonso Fonseca, Fulvio Alexandre Scorza, Carla Alessandra Scorza

**Affiliations:** ^1^ Disciplina de Neurociência, Departamento de Neurologia e Neurocirurgia, Universidade Federal de São Paulo (UNIFESP), São Paulo, Brazil.; ^2^ Laboratory of Nuclear Medicine (LIM43), Department of Radiology and Oncology, Faculdade de Medicina FMUSP, Universidade de Sao Paulo, São Paulo, Brazil; ^3^ Laboratório de Espectrometria de Massas—Associação Beneficente de Coleta de Sangue (COLSAN), São Paulo, Brazil; ^4^ Laboratory of Research, Development and Innovation, Apis Flora Indl, Coml, Ltda., São Paulo, Brazil; ^5^ Laboratório de Neurociências Experimental e Computacional, Departamento de Engenharia de Biossistemas, Universidade Federal de São João Del-Rei (UFSJ), Minas Gerais, Brazil; ^6^ Laboratório de Análises Clínicas da Faculdade de Medicina Do ABC, Santo André, São Paulo, Brazil; ^7^ Departamento de Ciencias Farmaceuticas da Universidade Federal de Sao Paulo (UNIFESP), Diadema, Brazil

**Keywords:** Parkinson’s disease, heart, propolis, 6-OHDA, metabolomics, [18F]FDG PET imaging

## Abstract

While there is sustained growth of the older population worldwide, ageing is a consistent risk factor for neurodegenerative diseases, such as Parkinson’s-disease (PD). Considered an emblematic movement disorder, PD comprises a miscellany of non-motor symptoms, for which effective management remains an unfulfilled need in clinical practice. Highlighted are the cardiovascular abnormalities, that cause significant burden in PD patients. Evidence suggests that key biological processes underlying PD pathophysiology can be modulated by diet-derived bioactive compounds, such as green propolis, a natural functional food with biological and pharmacological properties. The effects of propolis on cardiac affection associated to PD have received little coverage. In this study, a metabolomics approach and Positron Emission Tomography (PET) imaging were used to assess the metabolic response to diet supplementation with green propolis on heart outcomes of rats with Parkinsonism induced by 6-hydroxydopamine (6-OHDA rats). Untargeted metabolomics approach revealed four cardiac metabolites (2-hydroxybutyric acid, 3-hydroxybutyric acid, monoacylglycerol and alanine) that were significantly modified between animal groups (6-OHDA, 6-OHDA + Propolis and sham). Propolis-induced changes in the level of these cardiac metabolites suggest beneficial effects of diet intervention. From the metabolites affected, functional analysis identified changes in propanoate metabolism (a key carbohydrate metabolism related metabolic pathway), glucose-alanine cycle, protein and fatty acid biosynthesis, energy metabolism, glutathione metabolism and urea cycle. PET imaging detected higher glucose metabolism in the 17 areas of the left ventricle of all rats treated with propolis, substantially contrasting from those rats that did not consume propolis. Our results bring new insights into cardiac metabolic substrates and pathways involved in the mechanisms of the effects of propolis in experimental PD and provide potential novel targets for research in the quest for future therapeutic strategies.

## 1 Introduction

An alarming increase in the prevalence of age-related chronic diseases is predicted as people are living longer (Lisko et al., 2021). Parkinson’s disease (PD) is the second most frequent neurodegenerative disease and is growing rapidly, with a 2.5-fold increase in the number of cases in the last 30 years (Bloem et al., 2021; Pirazzini et al., 2021). This neurological condition was first described by James Parkinson in his remarkable 1817 study, when it was characterized by a clinical pattern of motor signs, just as it still is today (Del Rey et al., 2018). Although known as a typical movement disorder defined by the presence of bradykinesia, combined with resting tremor, and/or muscle rigidity caused by the loss of approximately 50% of dopaminergic neurons in the substantia nigra pars compacta, PD has several non-motor manifestations that usually occur in the prodromal phase of the disease, years before the diagnosis of PD (Durcan et al., 2019; Bloem et al., 2021; Longinetti et al., 2021). Autonomic disorders and cardiovascular abnormalities are among the most common non-motor symptoms of PD (Scorza et al., 2018; Park et al., 2020; Cuenca-Bermejo et al., 2021). It is expected that about 80%–90% of PD patients will have changes in the autonomic system, and a higher risk of dying due to major cardiac complications than the general population (Palma and Kaufmann, 2018; Scorza et al., 2018; Zhang and Chen, 2020; Gonçalves et al., 2021). Likewise, there is twice the prevalence of heart failure in patients with PD compared to the general population (Cuenca-Bermejo et al., 2021), and these patients are more susceptible to heart failure, coronary artery disease, sudden cardiac death, orthostatic hypotension, hypertension, bradycardia and reduced heart rate variability, pointing to direct changes in the sympathetic and parasympathetic nervous systems (Gonçalves et al., 2020; Finsterer et al., 2018). Therefore, the existence of heart disease needs to be recognized and monitored more intensively in PD, in order to improve the patient’s prognosis and quality of life. PD has no cure, and treatment focuses on motor symptoms with dopamine replacement drugs, starting after clinical diagnosis, even though non-motor symptoms are usually not controlled and may even be worsened by these therapies (Seppi et al., 2019). As a consequence, there is a crucial need for new therapeutic strategies.

A long tail of research findings pinpoint neuroinflammation, oxidative damage, immune disturbances, mitochondrial dysfunction and energy disturbances as important features of the complex physiopathological processes underlying PD as well as cardiovascular diseases (Simon et al., 2019; Sian-Hulsmann and Riederer, 2021). Interestingly, it has been shown that these key biochemical events may suffer nutritional influences, suggesting a role for nutrition interventions in targeting these processes for disease management. Propolis, a natural substance produced by *Apis mellifera* bees and used for centuries for human well-being (Kuropatnicki et al., 2013; Berretta et al., 2017), represents an example of substance with a variety of pharmacological and biological properties which can, potentially be used as a drug (Sforcin and Bankova, 2011), namely anti-inflammatory (Paulino et al., 2008; Hori et al., 2013), antioxidant (Kuropatnicki et al., 2013; Diniz et al., 2020) and immunoregulatory activities (Sforcin, 2007; Machado et al., 2012), among several others, justifying its classification as a functional food (Berretta et al., 2017; Irigoiti et al., 2021). Therefore, propolis supplementation has been assessed in several studies as a countermeasure to the underlying pathological processes associated with diseases, showing beneficial activities in cardiovascular and neurological disorders such as PD (Przybyłek and Karpiński, 2019; Ali and Kunugi, 2020; Gonçalves et al., 2020; Bhargava et al., 2021). Bees produce propolis from different botanical sources to which they mix their saliva, wax and pollen and its chemical composition is rich in flavonoids, phenolic and chlorogenic acids, terpenoids and phenylpropanoids, esters, fatty acids, amino acids, polysaccharides, cinnamic acid derivatives, vitamins and minerals, and other biologically active phytonutrients (Przybyłek and Karpiński, 2019; Sun et al., 2019; Bhargava et al., 2021). To provide valuable context when assessing the impact of nutritional interventions, untargeted metabolomics allows to scan metabolites in biological samples, such as heart tissue, and uses statistical metrics to reveal differences of specific compounds and diverse metabolic pathways that reflect metabolic responses to dietary supplements. Positron emission tomography (PET) with 18 F-2-fluoro-2-deoxy-d-glucose ([18F]FDG) represents a worthy tool for *in vivo* assessment of the cardiac metabolic rate of glucose.

Nevertheless, our understanding of how the heart itself is affected in PD is still rudimentary. The lack of access to the human brain and heart limits the study of these factors, so the use of animal models that mimic different aspects of the disease is essential. Intracerebral injection of the neurotoxin 6-OHDA is widely used in PD research due to consistent results in predictable degeneration of dopaminergic neurons. The 6-OHDA model also mimics several non-motor features of disease often detected in patients, including cardiovascular manifestations (Silva et al., 2015; Rodrigues et al., 2019; Gonçalves et al., 2020; Masini et al., 2021) thus, providing an important platform for assessing potential therapeutic interventions.

In this study, the effects of standardized green propolis extract (EPP-AF^®^) intervention on heart outcomes of parkinsonian rats were investigated through PET imaging [18F]FDG to assess cardiac 2-Deoxy-2-18F-fluoro-d-glucose (18F-FDG) uptake and by metabolomics, which offers a broad view of the metabolic response to dietary propolis supplementation by measuring and detecting cardiac tissular metabolites and related metabolic pathways.

## 2 Materials and methods

### 2.1 Animals

The experimental procedures were performed according to the guidelines established by the Ethics Committee of the Federal University of São Paulo (CEUA number 4260280521), and every effort was made to minimize the suffering of the animals. A total of 32 adult male Wistar rats weighing between 230–300 g and 8 weeks of age were used. The animals came from the central vivarium of the Federal University of São Paulo and were kept in the animal vivarium of the neuroscience laboratory, housed in groups of four per cage with appropriate sawdust and free access to water and feed under a light-dark cycle (light: 7:00–19:00) and constant temperature of 21 ± 2°C.

### 2.2 Study design

The animals were randomly divided into four different groups, in which the ending + P stands for propolis: sham, sham + P, 6-OHDA and 6-OHDA + P. Animals in the 6-OHDA groups received a bilateral injection of a 6-hydroxydopamine neurotoxin into the striatum. The animals in the sham groups, that received filtered water or propolis, underwent the same surgical procedures in the brain points, however, only injected with saline solution. Twenty-four hours after the stereotaxic surgery, the animals in the +P groups received daily gavage with Brazilian green propolis (200 mg/kg) (Arabameri et al., 2017; Takeda et al., 2018; Gonçalves et al., 2020), while the rats in the other groups received gavage with filtered water. After 40 days of treatment, the animals were submitted to PET imaging to assess cardiac [18F]FDG uptake. At the end of the procedures, the animals were euthanized and the brains and hearts were removed for immunohistochemistry and metabolomics, respectively.

### 2.3 6-OHDA lesion

Fifteen minutes before stereotactic surgery, the animals received intraperitoneal injections of ketamine (100 mg/kg) and xylazine (10 mg/kg) and after complete anesthesia, they were fixed in a stereotaxic device (EFF 331-Insight™, Ribeirão Preto, São Paulo, Brazil). A 10 µl Hamilton syringe attached to the stereotaxic rod was used to inject 1 µl of 6-OHDA solution (Sigma^©^, Saint Louis, MO, United States) (12 μg/μl concentration in 0.3% ascorbic acid) or saline solution in four different coordinates: 1) laterolateral: −2.7 mm, anteroposterior: bregma, dorsoventral: −4.5 mm; 2) latero-lateral: −3.2 mm, anteroposterior: +0.5 mm, dorsoventral: −4.5 mm; 3) latero-lateral: +2.7 mm, anteroposterior: bregma, dorsoventral: −4.5 mm; and 4) latero-lateral: +3.2 mm, anteroposterior: +0.5 mm, dorsoventral: −4.5 mm, according to the rat brain atlas by Paxinos and Watson (2009). Bilateral administration of 6-OHDA, a catecholamine selective neurotoxin, to the dorsal striatum retrogradely causes cell death of neurons containing tyrosine hydroxylase (TH) in the substantia nigra pars compacta (SNc) mimicking Parkinson-like pathology (Shimohama et al., 2003; Perlbarg et al., 2018).

### 2.4 Propolis gavage

One day after the surgical procedures, the propolis solution (200 mg/kg) was administered by daily gavage to sham + P and 6-OHDA + P animals and filtered water was given to the remaining groups. The new version of Brazilian green propolis standardized extract (EPP-AF®-C, Apis Flora, Ribeirão Preto, São Paulo, Brazil) was presented in a powder developed using a dryness process, using spray-dryer technology according to the process previously published by Marquiafável et al. (2015) with some modifications, as tapioca modified starch as excipient. The propolis powder was evaluated and characterized using HPLC-DAD with C18-reverse column methodology as previously published by Berretta et al. (2012). Total phenolic and total flavonoids were measured by spectrophotometric methods, using Folin-Ciocalteu and aluminium chloride complexation, as published by Woisky and Salatino (1998), using galangin (Sigma-Aldrich, L. BCCG2648) and rutin (Phytolab, L. 66853802) as reference standards. Caffeic acid and galangin was acquired from Sigma-Aldrich (L. SLBZ6416 and BCCG2648, respectively), 3,5-dicaffeoylquinic acid, 4,5- dicaffeoylquinic acid and artepellin C were purchase from Phytolab (L. 3215, 9943 and L. 111674647, respectively), aromadendrin-4′-O-methyl ether, drupanin and baccharin were previously isolated and identified by (Sousa et al. (2009) and kindly donated by the authors.

### 2.5 PET scan

**FIGURE 1:**
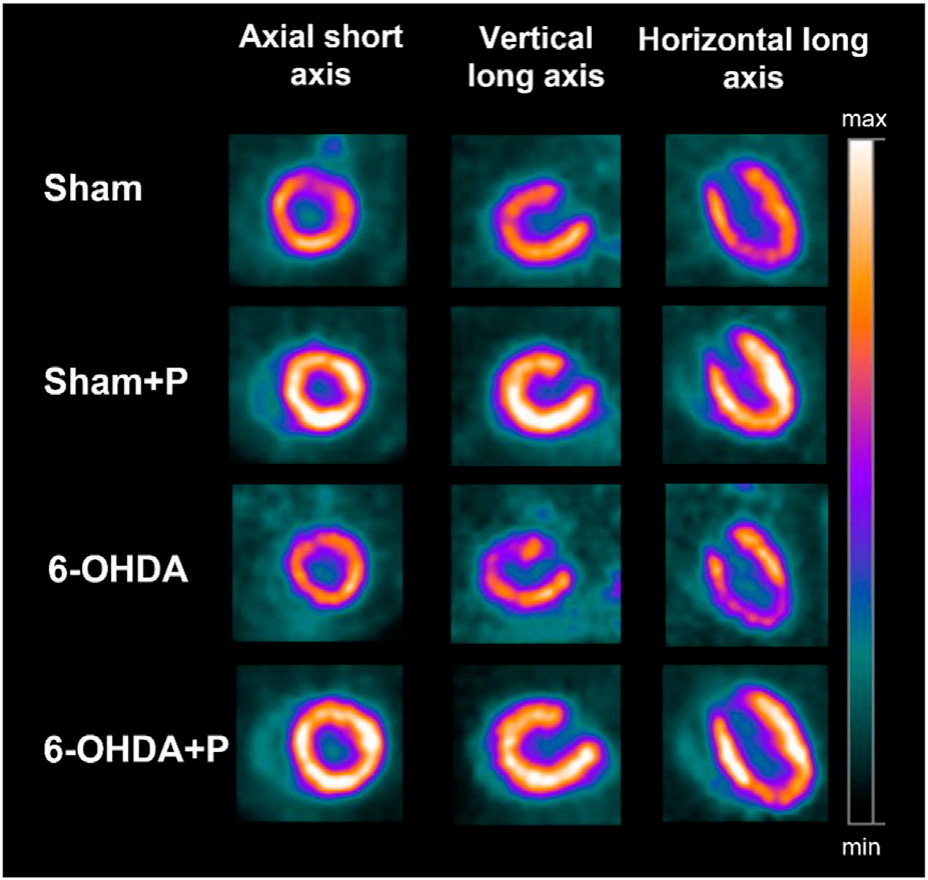
Illustrative [18F]FDG PET images showing the three cardiac axes (columns) of the different groups (lines). Note that the [18F]FDG uptake is higher in the sham + P and 6-OHDA + P groups.

**FIGURE 2:**
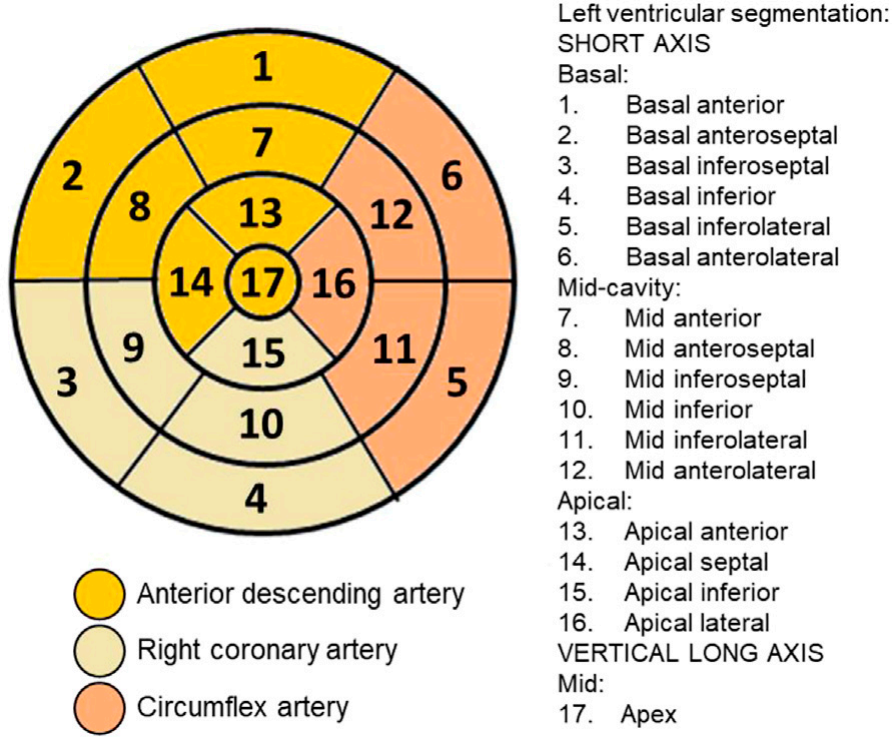
Plot of segments and coronary arterial territories: Polar map of the 17 segments that divide the left ventricle and their correspondence with the coronary territories for use in cardiac imaging techniques, defined by the American Heart Association.

PET images were acquired with radiopharmaceutical [18F]FDG (*n* = 8/group) in a specific equipment for small animals (Triumph™ - Gamma Medica-Ideas, Northridge, CA, United States) and the rats were anesthetized with 2%–3% isoflurane in 100% oxygen throughout the procedure. The blood glucose of the animals was measured and then, ±1 mCi (37 MBq) of [18F]FDG was administered intravenously in the penile vein. The thoracic (heart in the center of the field of view) image acquisition started 45 min after [18F]FDG administration and lasted 10 min. The images were reconstructed with OSEM-3D algorithm, using 20 iterations and four subsets. Figure 1 shows [18F]FDG PET illustrative images. The PET images were analyzed in the PMOD TM software (Swtizerland), using the cardiac module (PCARDIO). In cardiac images, the results are presented according to the 17-segment polar map (Figure 2) available in the software. Results are presented in SUV (Standardized Uptake Value), which is the quantification of the radioactive substance in a given volume, corrected for the injected dose and the animal’s body mass.

### 2.6 Immunohistochemistry

Tyrosine hydroxylase immunostaining was performed to confirm the death of nigrostriatal dopaminergic cells in the 6-OHDA-treated rats, as well as the neuroprotective effects of propolis to dopaminergic neurons, as previously described (Gonçalves et al., 2020). Animals were anesthetized intraperitoneally with lidocaine (10 mg/kg) and sodium thiopental (80 mg/kg). After complete analgesia, they were euthanized for decapitation using a guillotine. The brains were removed and those that would be used for immunohistochemistry were placed in 0.01 M phosphate buffered saline (PBS) (pH 7.4) and the hearts that would be used for metabolomics were removed and immersed in liquid nitrogen and then stored in a freezer at −80°C until use. Afterwards, the brains were immersed in 30% sucrose cryoprotectant solution for 48 h to be cut (40 μΜ coronal sections) using a cryostat (Microm HM 505E). Brain tissues were bathed three times in 0.01 M PBS (pH 7.4), treated with 0.1% hydrogen peroxide and, after washing, incubated in 10% albumin solution and 0.3% Triton X-100. for 2 h. Slices were incubated overnight with primary antibody (1:500 tyrosine hydroxylase (TH), diluted in 0.01 M PBS (pH 7.4) and 2% albumin. After three washes with 0.01 M PBS (pH 7.4), slices were treated with biotinylated secondary antibody (anti-rabbit 1:200-Abcam^®^) diluted in 0.01 M PBS and 2% albumin, washed and incubated with avidinbiotin–peroxidase complex (ABC Elite; Vector Labs, Burlingame, CA, United States). Next, sections were stained with 3,3′-diaminobenzidine (DAB) tetrachloride dissolved in 0.05 M TRIS–HCl (PH 7.6) activated by 0.3% hydrogen peroxide. The slices were placed on silanized slides, subjected to the dehydration and diaphanization process, and the slides were photographed in an apparatus. The images were analyzed by the ImageJ program to indicate the objects of interest by positive immunoreactivity.

### 2.7 Metabolomics

In the processing step, the hearts were removed from the −80°C freezer and homogenized in a suitable solution for lysis (TissueLyser; Qiagen, Germantown, MD). For GC-MS analysis, 100 uL aliquots of sample from each group will be vortexed with pure methanol at 4°C in a 1:3 ratio for deproteinization to occur, and centrifuged at high speed. 100 uL of the supernatant will be transferred to GC-MS vials containing glass inserts for the derivatization process. For the methoximation step, the solvent must be evaporated at 30°C in a SpeedVac and the O-methoxyamine hydrochloride (15 mg/ml) in pyridine added to the vials, vortexed and incubated for 16 h in the dark at room temperature. After this period, silylation begins with 10 uL of BSTFA [1% TMCS (v/v)] and samples are incubated at 70°C for 1 h. Finally, 100 uL of pentadecanoic acid (20 ppm in heptane) will be added to each analysis vial. Blanks were be prepared and analyzed together to correct the baseline of the chromatograms (Mastrangelo et al., 2015). The analyzes will be carried out in a quadrupole-type GCMS-QP2020NX system (Shimadzu Co., Kyoto, Japan), with 1 uL of the sample loaded into a DB5-MS column (30 m × 0.25 mm, 0.25 µm, Restek) and injected in splitless mode in a total flow of 20 ml/min of helium gas. Carrier gas will be conducted at a constant flow of 1.36 ml/min. The initial column temperature will be initially maintained at 100°C and then gradually increased at a rate of 15°C/min until reaching the final temperature of 300°C and maintained at this temperature for 5 minutes before cooling it. The temperatures of the injector, transfer line and source filament and the quadrupole will be maintained at 280°C, 200°C, 150°C, respectively. The system will operate in full scan mode (*m*/*z* 40–650) at a rate of three spectra/s, and with the EI set to 70 eV. Instrument control, data acquisition and data processing performed by LabSolutions software (GCMS version 4.5, Shimadzu Co., Japan), which allows real-time control of each analyzed analyte for the identification of metabolites in SIM and Scan. For the analysis in Scan mode, the detected metabolites will be processed to create a unified matrix of variables from the different states of charge, adducts and groups of the same analytes from all samples using the GCMS Solution software (v.3.30), NIST 17MASS (v.1.00.1) and GCMS Smart Metabolite (v.3.01), all developed by Shimadzu Co. After the identification of the molecules by the NIST [14] and Smart Metabolite libraries, the samples will be exported to the Excel software (Microsoft Office) for statistical treatment using the available free online tool metaboanalyst 5.0. Public databases available on the internet, like the Human Metabolome Database (https://hmdb.ca/) were used (www.metlin.scripps.edu, https://www.genome.jp/kegg/, www.lipidmaps.org or http://www.hmdb.ca) which may also be used to identify the fragmentation profiles.

### 2.8 Statistics

Immunohistochemistry and PET data were not derived from a normal distribution, tested by the Kolmogorov-Smirnov test. Therefore, the non-parametric Kruskal–Wallis test was used to simultaneously compare the four groups studied. The Bonferroni post hoc test determined which groups were significantly different from the others. Metabolomics data were normalized, the parametric ANOVA test was used to simultaneously compare the four groups. Tukey’s post hoc test determined which groups were significantly different from the others. The statistical significance level was set at 0.05. Statistical tests were performed using MATLAB R2017a and Metaboanalyst 5.0.

## 3 Results

The Brazilian green propolis extract used EPP-AF®-C, presented 38.27% of total phenolic and 8.69% of total flavonoid expressed as galangin and rutin, respectively. The chemical fingerprint of the extract used was obtained by HPLC/DAD and it is presented in the Supplementary Material. Quantitative results demonstrated that each gram of the powder used presented 2.04 mg of caffeic acid, 3.21 mg of 3,5-dicaffeoylquinic acid, 1.71 mg of 4,5-dicaffeoylquinic acid, 3.88 mg of aromadendrin-O′-methyl ether, 10.04 mg of chrysin, 7.30 mg of galangin, 9.12 mg of drupanin, 24.30 of artepellin C and 2.65 mg of baccharin.

### 3.1 Tyrosine hydroxylase immunohistochemistry

**FIGURE 3:**
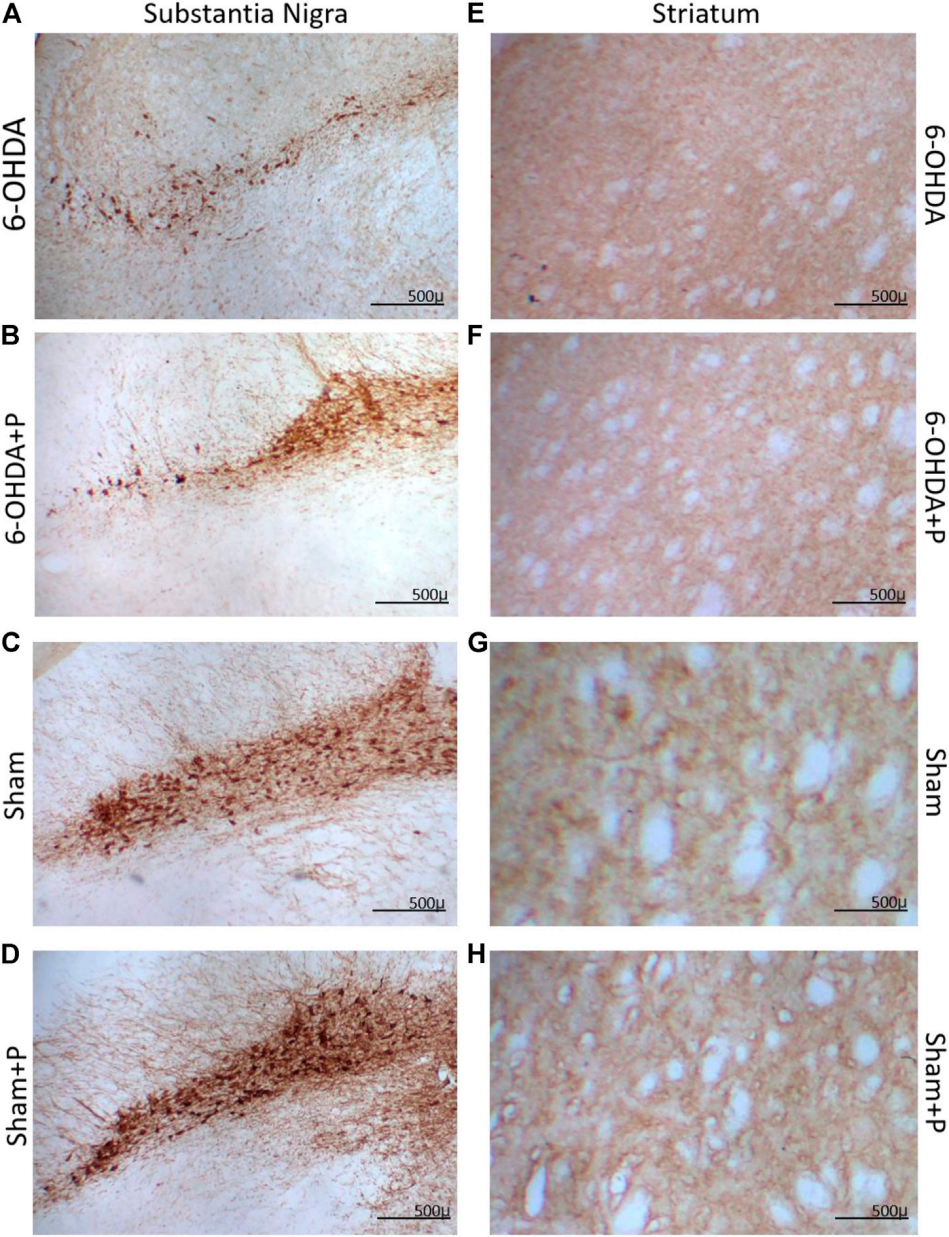
Illustrative images of tyrosine hydroxylase (TH) immunoreactive sections. Dopaminergic neurons in the substantia nigra pars compacta in **(A)** 6-OHDA; **(B)** 6-OHDA + P; **(C)** Sham; **(D)** Sham + P. Striatal fibers in **(E)** 6-OHDA; **(F)** 6-OHDA + P; **(G)** Sham; **(H)** Sham + P. Note stronger TH-positive staining in the propolis-treated parkinsonian rats **(B,F)** in comparison to sham **(A,E)**.

Eight animals (*n* = 8) from each group were tested for TH-positive immunoreactivity, used as a marker of viable dopaminergic neurons in the substantia nigra pars compacta and dopaminergic fibers in the striatum (Figure 3).

**FIGURE 4:**
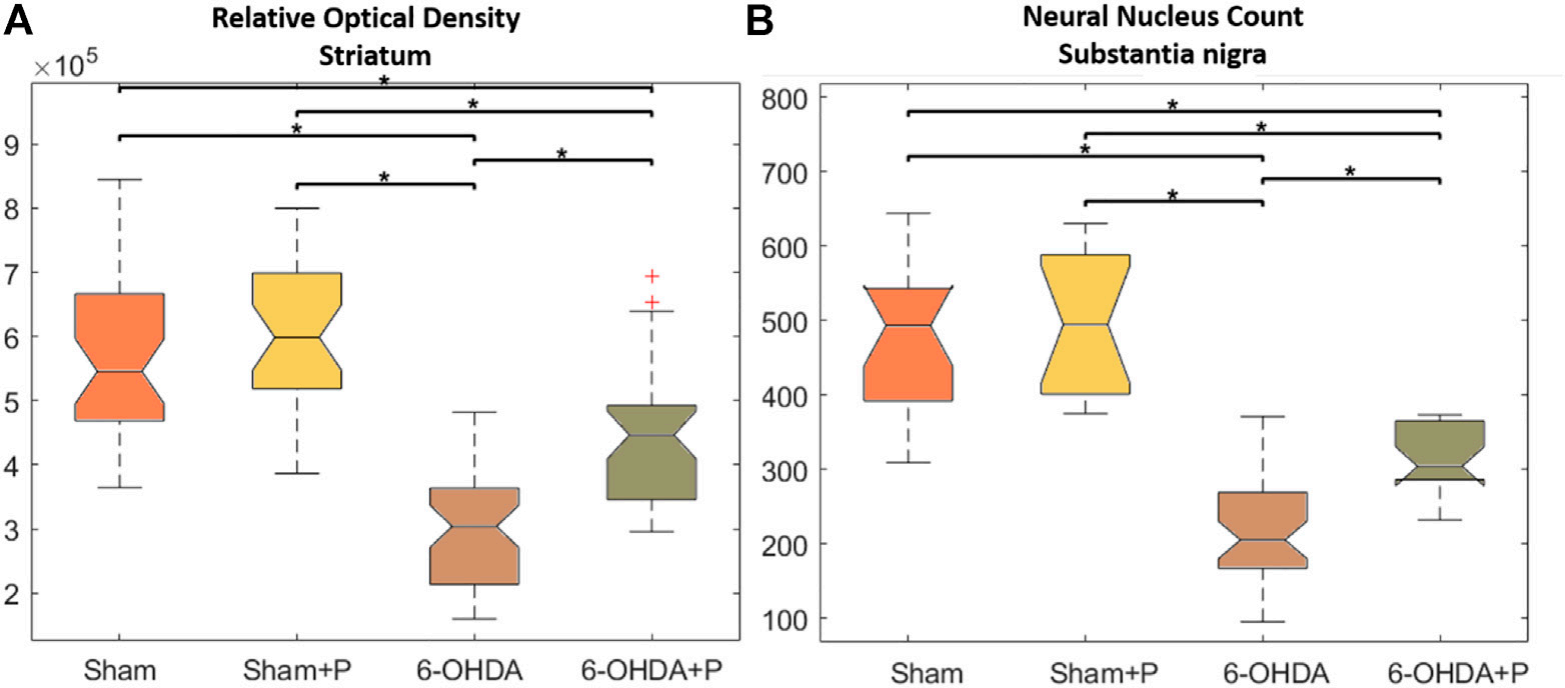
Statistical results of the Kruskall-Wallis and post hoc Bonferroni for TH immunohistochemistry analysis. **(A)** Optical density of the TH-positive fibers in the striatum; median and confidence interval were as follows: 5.458 × 10 + 5 ± 2.5694 × 10 + 4 (sham); 5.984 × 10 + 5 ± 2.862 × 10 + 4 (sham + P); 4.466 × 10 + 5 ± 1.908 × 10 + 4 (6-OHDA + P); 3.04 × 10 + 5 ± 1.451 × 10 + 4 (6-OHDA). **(B)** TH-positive neuronal nuclei in the substantia nigra pars compacta; median and confidence interval were as follows: 493 ± 54 (sham); 494 ± 78 (sham + P); 205 ± 26 (6-OHDA + P); 304 ± 25 (6-OHDA).

In the striatum region, the relative optical density approach showed that all groups were significantly different from the 6-OHDA group (Figure 4A). Through quantification of the dopaminergic neuronal nuclei in the substantia nigra pars compacta, significant differences were detected in all groups when compared to the 6-OHDA group (Figure 4B). In both situations, there was no significant difference between the Sham and Sham + P groups, however, a significant difference was found between these groups and the 6-OHDA + P group. 6-OHDA-injured rats exhibited lower fiber density in the striatum and fewer TH-positive neural nuclei than propolis-treated 6-OHDA-injured rats, suggesting a protective effect of propolis against 6-OHDA-induced neurodegeneration of the nigrostriatal dopaminergic pathway. Our results showed that 6-OHDA injection resulted in an average 58% reduction in dopaminergic neuronal nuclei in the substantia nigra pars compacta area and an average 44% reduction in striatal fibers compared to the control group. In contrast, 6-OHDA rats treated with propolis showed a mean neuronal loss of 38% in the substantia nigra pars compacta and a mean decrease of 19% in striatal fiber density compared to the control group.

### 3.2 [18F]FDG Positron Emission Tomography imaging

**FIGURE 5:**
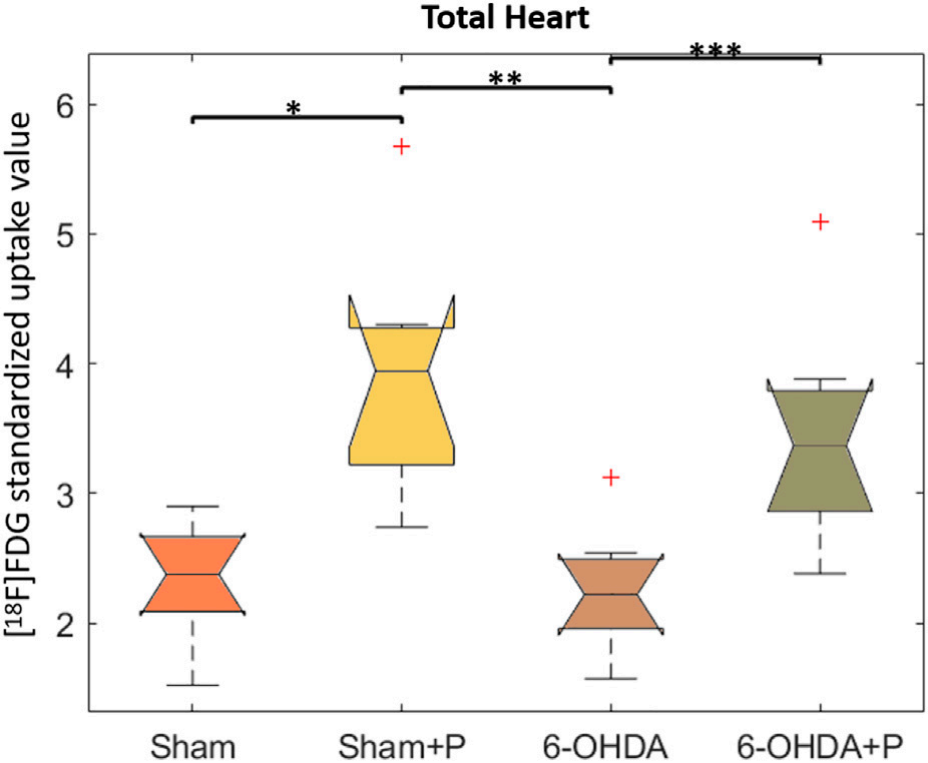
Standardized [18F]FDG uptake value in left ventricle. Comparison between the Sham, Sham + P, 6-OHDA and 6-OHDA + P. *p = 0.005; **p = 0.003; ***p = 0.01.

The analysis was performed on 31 animals (groups: sham, *n* = 8; sham + P, *n* = 8; 6-OHDA, *n* = 8; 6-OHDA + *p*, *n* = 7). The [18F]FDG PET imaging data are described in Table 1 and show, in all segments of the left ventricle, increased values in glucose metabolism in the +P groups, regardless of whether they are from the Sham or 6-OHDA group (Figure 5).

**TABLE 1:** Median data and confidence interval of [18F]FDG uptake value in different areas of the left ventricle.

Coronaryterritory	Sham	Sham + p	6-OHDA	6-OHDA + p
Total heart	2.37 ± 0.32	3.94 ± 0.59	2.28 ± 0.28	3.42 ± 0.52
Basal anterior	2.58 ± 0.28	4.55 ± 0.52	2.62 ± 0.28	3.64 ± 0.57
Basal anteroseptal	2.61 ± 0.31	3.93 ± 0.43	2.55 ± 0.22	3.53 ± 0.39
Basal inferoseptal	2.37 ± 0.22	3.96 ± 0.31	2.34 ± 0.22	3.58 ± 0.26
Basal inferior	2.26 ± 0.19	3.64 ± 0.58	2.14 ± 0.19	3.17 ± 0.19
Basal inferolateral	2.21 ± 0.22	3.63 ± 0.63	2.16 ± 0,30	3.06 ± 0.51
Basal anterolateral	2.41 ± 0.23	4.05 ± 0.67	2.34 ± 0.24	3.52 ± 0.48
Mid anterior	2.74 ± 0.35	4.44 ± 0.57	2,73 ± 0.24	4.18 ± 0.66
Mid anteroseptal	2.57 ± 0.34	4.05 ± 0.5	2.49 ± 0.28	3.86 ± 0.66
Mid inferoseptal	2.51 ± 0.36	3.94 ± 0.64	2.24 ± 0.34	3.61 ± 0.51
Mid inferior	2.30 ± 0.30	3.83 ± 0.61	1.95 ± 0.28	3.29 ± 0.45
Mid inferolateral	2.19 ± 0.35	3.99 ± 0.82	2.00 ± 0.29	3.56 ± 0.63
Mid anterolateral	2.53 ± 0.41	4.07 ± 0.68	2.44 ± 0.34	3.94 ± 0.64
Apical anterior	2.50 ± 0.50	4.20 ± 0.78	2.58 ± 0.38	3.70 ± 0.35
Apical septal	2.33 ± 0.40	3.68 ± 0.62	2.25 ± 0.17	3.53 ± 0.63
Apical inferior	2.16 ± 0.3	3.4 ± 0.50	1.96 ± 0.2	3.14 ± 0.44
Apical Lateral	2.28 ± 0.39	3.82 ± 0.52	2.19 ± 0.25	3.33 ± 0.3
Apex	2.12 ± 0.26	3.39 ± 0.47	1.96 ± 0.18	2.94 ± 0.32

### 3.3 Untarget metabolomics analyzes on heart tissue samples

**FIGURE 6:**
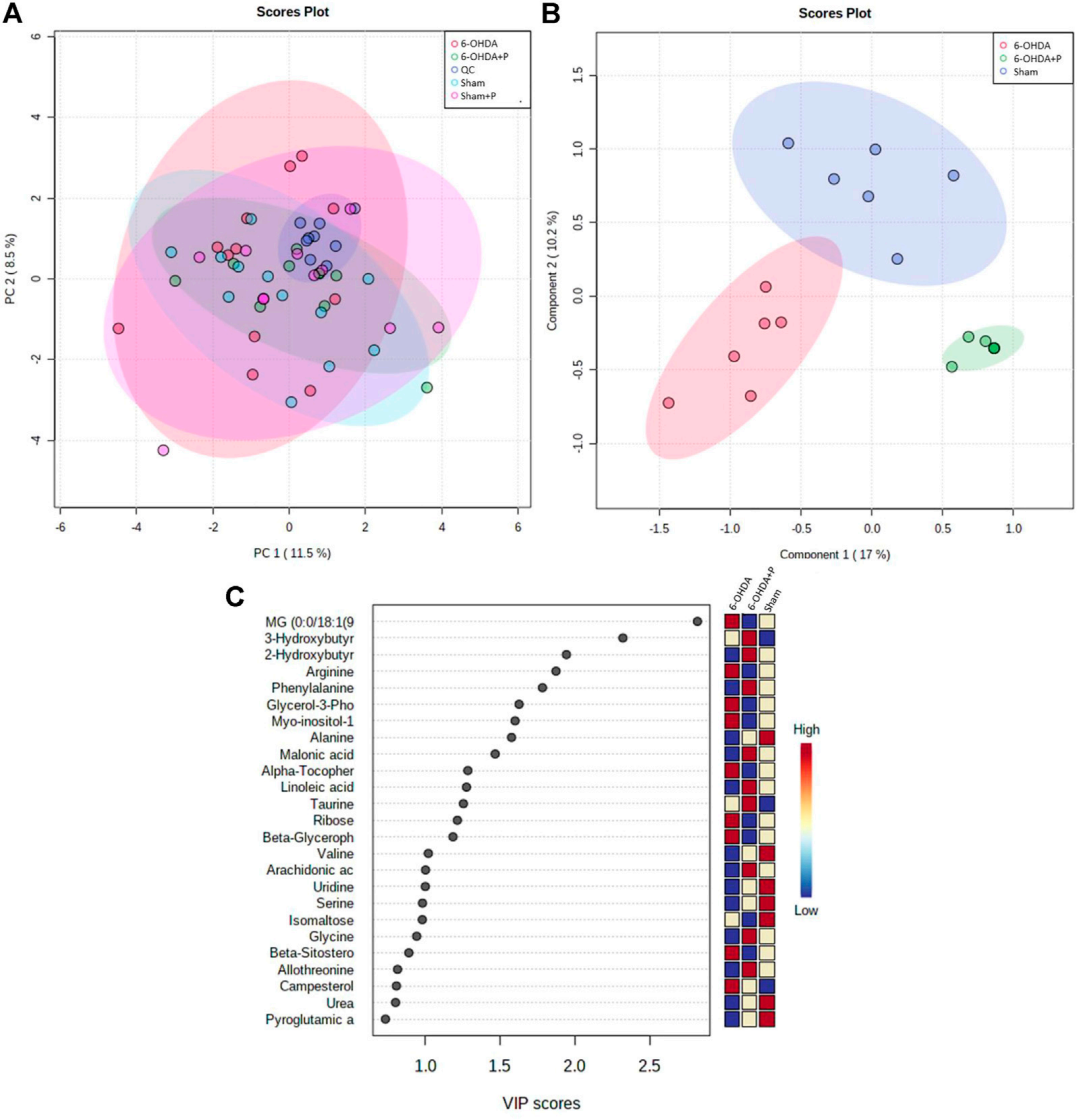
Major metabolites present in rat heart tissue. Multivariate statistical analysis between the 6-OHDA, 6-OHDA + P and Sham groups and the main metabolites found. **(A)** PCA analysis to investigate the interrelationships between the most important variables (PC1, main component one; PC2, main component two) found and to verify the reliability of the data between the groups. **(B)** PLS plotting with the separation of groups in relation to the metabolites found. **(C)** List of the main metabolites identified in the heart of animals, shown as a VIP score. The colored boxes on the right indicate the relative concentrations of the corresponding metabolite for the control animals and the 6-OHDA-lesioned animals that consumed propolis or not.

The metabolomics-based approach performed on eight animals per group offers a comprehensive view of the cardiac metabolic responses to parkinsonism and propolis consumption. After identifying all the metabolites present, multidimensional analyzes [Principal Component Analysis (PCA) (Figure 6A) and Partial Least Squares (PLS) (Figure 6B)] showed that the technique is robust, the samples are not biased and the groups are separated according to the main features. The Sham and Sham + P groups proved to be closely correlated, and as a consequence the Sham + P group was not considered in further analyses. The metabolites correlated to the 6-OHDA, 6-OHDA + P and Sham groups were hierarchically scaled in a Variable Importance Projection (VIP) score (Figure 6C).

**FIGURE 7:**
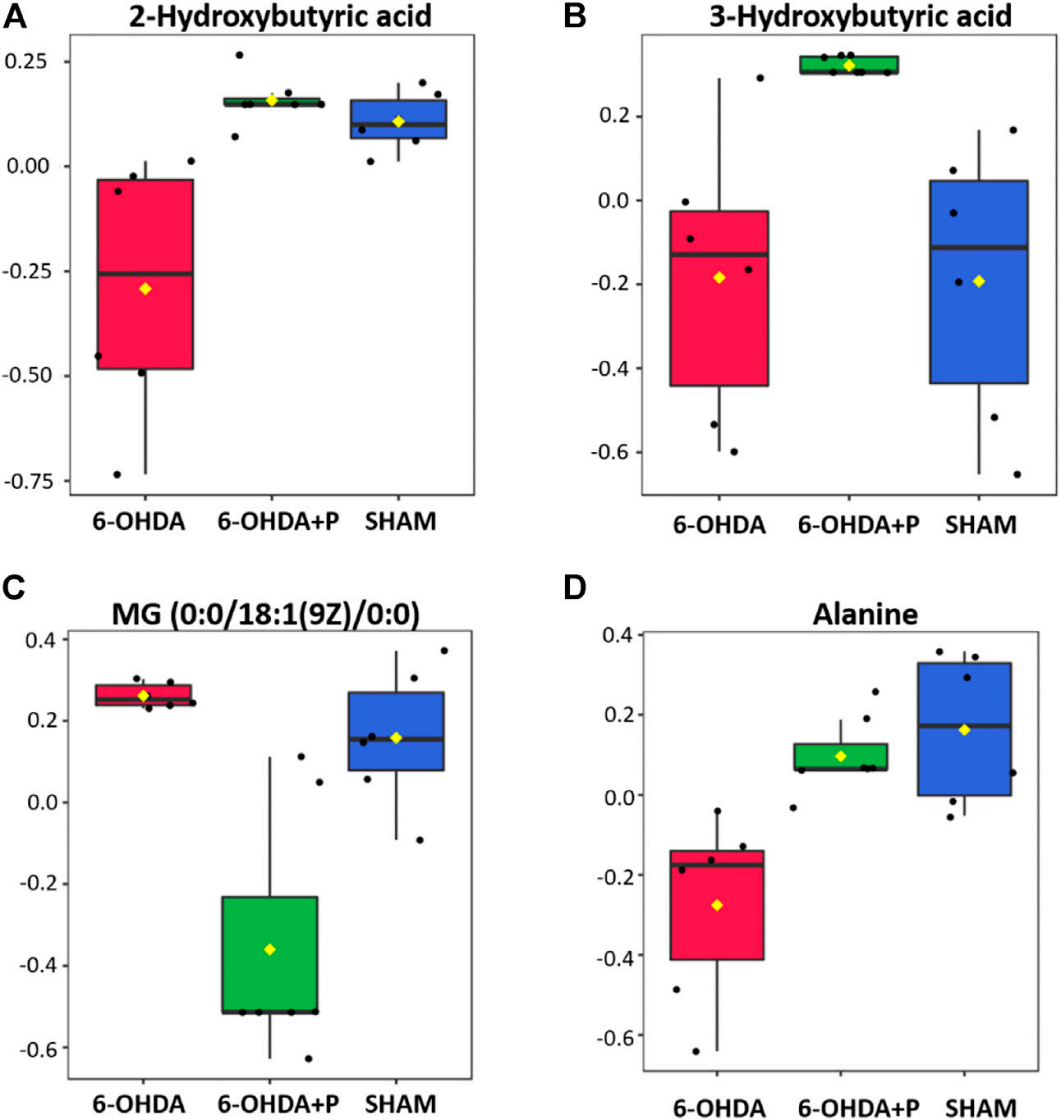
Statistical analysis of the four metabolites with the highest estimated amounts in the comparison between groups. **(A)** Estimated amounts of the 2-hydroxubutyric acid metabolite in the 6-OHDA, 6-OHDA + P and Sham groups. **(B)** Estimated amounts of the 3-hydroxubutyric acid metabolite in the 6-OHDA, 6-OHDA + P and Sham groups. **(C)** Estimated amounts of the MG (0:0/18:1 (9Z)/0:0) metabolite in the 6-OHDA, 6-OHDA + P and Sham groups. **(D)** Estimated amounts of the Alanine metabolite in the 6-OHDA, 6-OHDA + P and Sham groups.

The highest amounts of metabolites, present simultaneously in the 6-OHDA, 6-OHDA + P and Sham groups, were compared to determine whether there were significant differences (FDR adjusted to *p*-value < 0.05) in the concentration of these metabolites in each group (Table 2). Statistical analysis exposed significant changes in four metabolites levels, then, the graphs were plotted to better visualize the differences in the concentrations. The 2-Hydroxybutyric acid (Figure 7A) presents higher concentration in the 6-OHDA + P and Sham groups, and both are significantly different when compared to the 6-OHDA group. 3-Hydroxybutyric acid (Figure 7B) presents higher concentration in the 6-OHDA + P group, and its level is significantly different when compared to both 6-OHDA and Sham group. Monoacylglyceride (MG) (0:0/18:1 (9Z)/0:0) (Figure 7C) presents lower concentration in the 6-OHDA + P group, and its level is significantly different when compared to both 6-OHDA and Sham group. The metabolite Alanine (Figure 7D) presents higher concentration in the 6-OHDA + P and Sham groups, and both are significantly different when compared to the 6-OHDA group.

**TABLE 2:** Table with metabolites with statistically significant difference within groups, indicated by Tukey’s HSD test.

Metabolites	*p* value	FDR	Tukey’s HSD
MG (0:0/18:1 (94/0:0)	0.00012005	0.0033615	Park + Prop-Park, Sham-Park + Prop
2-Hydroxybutyric acid	0.00081041	0.015128	Park + Prop-Park, Sham-Park
Alanine	0.001129	0.015806	Park + Prop-Park, Sham-Park
3-Hydroxybutyric acid	0.0033589	0.03762	Park + Prop-Park, Sham-Park + Prop

**FIGURE 8:**
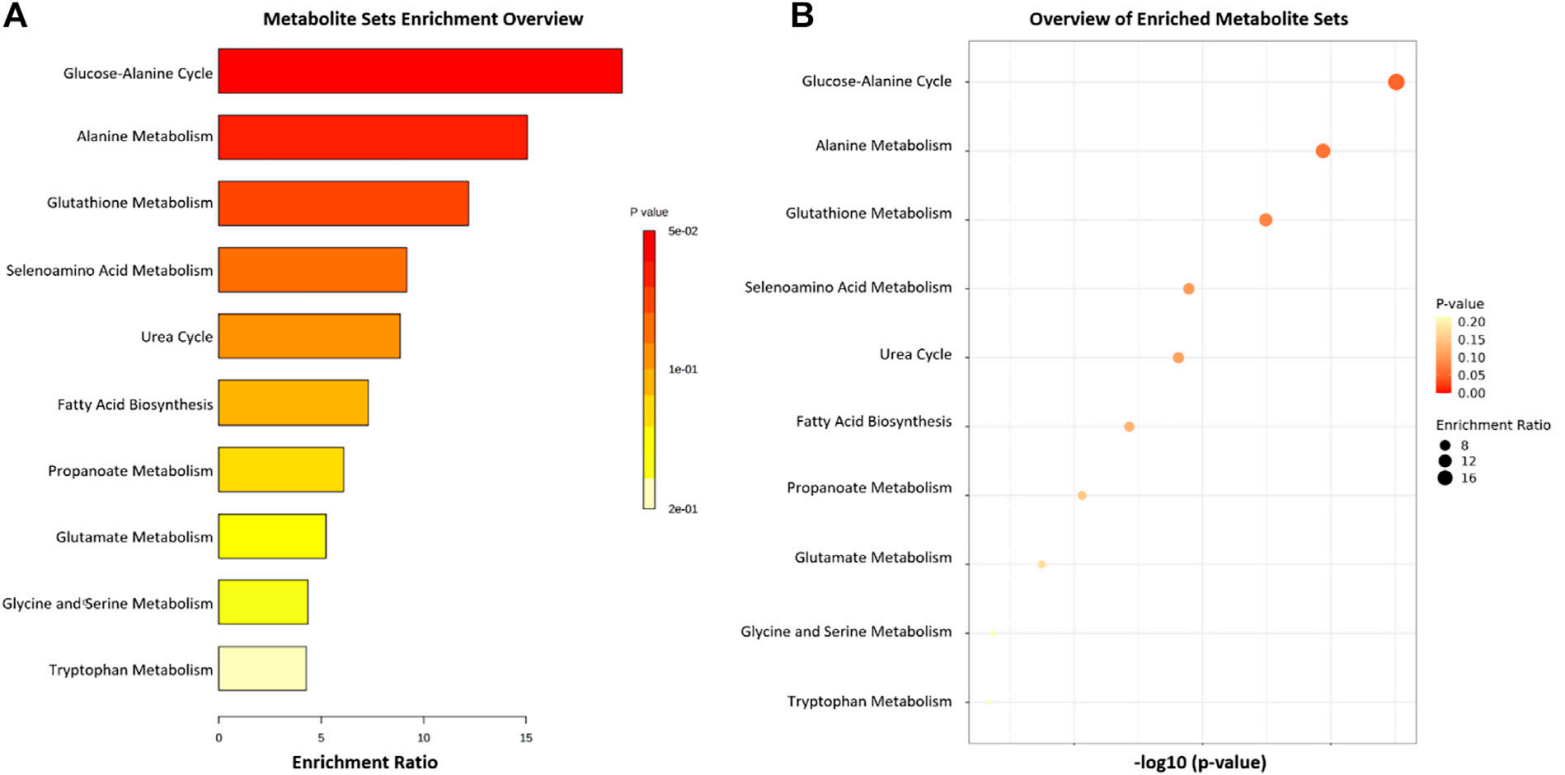
List of the 11 main pathways related to the four metabolites compared between the 6-OHDA, 6-OHDA + P and Sham groups. **(A,B)** Metabolite enrichment analysis indicating major metabolic pathways involved.

The Metaboanalyst 5.0 application was used to indicate the pathways associated with the metabolites found in higher concentration in the groups studied, identified by the color and ratio size (Figure 8). The main pathways listed (Propanoate Metabolism, Glucose-Alanine Cycle, Alanine Metabolism, Glutathione Metabolism and Urea cycle), with greater participation and relationship with the studied metabolites, are directly associated with energy control and regulation of cellular oxidative stress, crucial functions for the development of PD, as well as cardiac alterations. Propolis appeared to act directly on these pathways, mitigating the neurological and cardiac effects of PD.

## 4 Discussion

**FIGURE 9:**
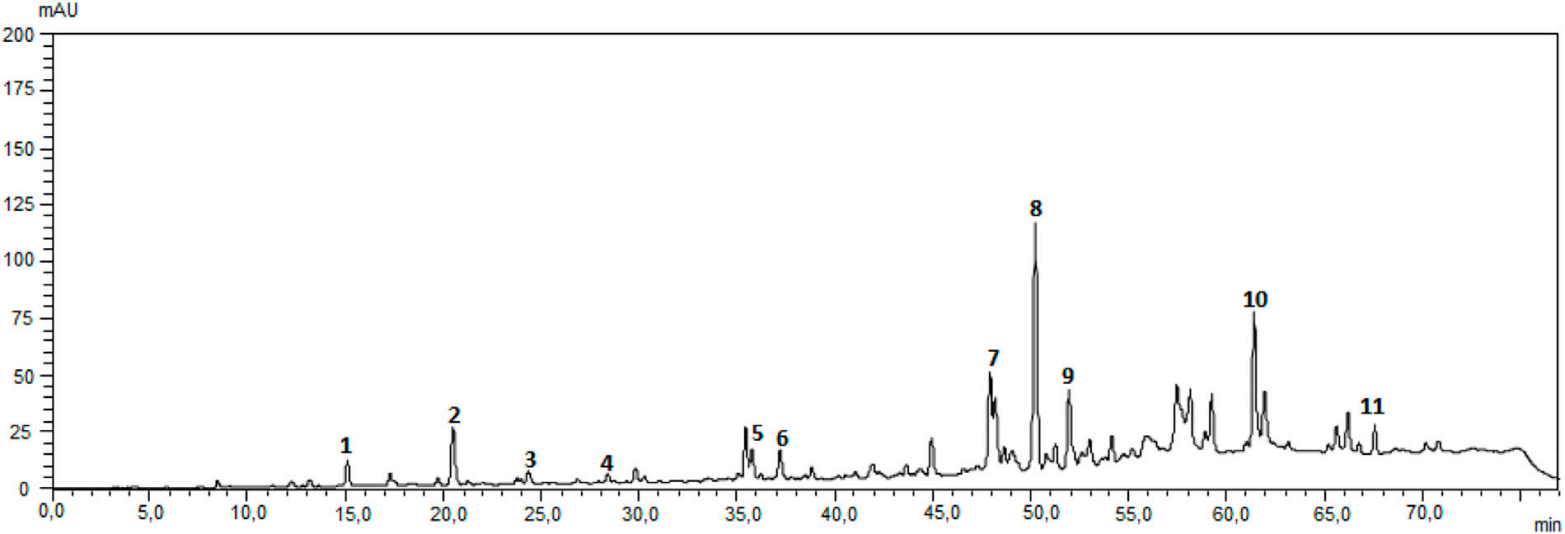
Chromatographic fingerprint of Propolis EPP-AF(R)-C obtained using a Shimadzu HPLC system, with LC-20AT quaternary delivery system, equipped with an autosampler, a CTO-10AC column oven, and a DAD-SPD-M20 A photodiode array detector, reverse-phase Shim Pack CLC-ODS (C18) was used analytical column (250 mm × 4.6 i.d, and a particle size of 5 um) from Shimazu and a pre-column was also used. The mobile phase consisted of a linear gradient of MiliQ acidified water with 0.1% of formic acid (pH 2.7) **(A)** and metanol **(B)**, from 20% to 80% of B, in 70 min (Berretta et al., 2012). The wavelength used was 275 nm and the flow rate was 0.8 ml/min. Authenticated standard were used for the identification of the peaks, that were quantified with an analytical curve for each. (1) caffeic acid, (2) p-coumaric acid, (3) 3,5-dicaffeoylquinic acid, (4) 4,5-dicaffeoylquinic acid, (5) cinnamic acid, (6) aromadendrin-4′-O-methyl ether, (7) drupanin, (8) crysin, (9) galangin, (10) artepellin C, and (11) baccharin.

**FIGURE 10:**
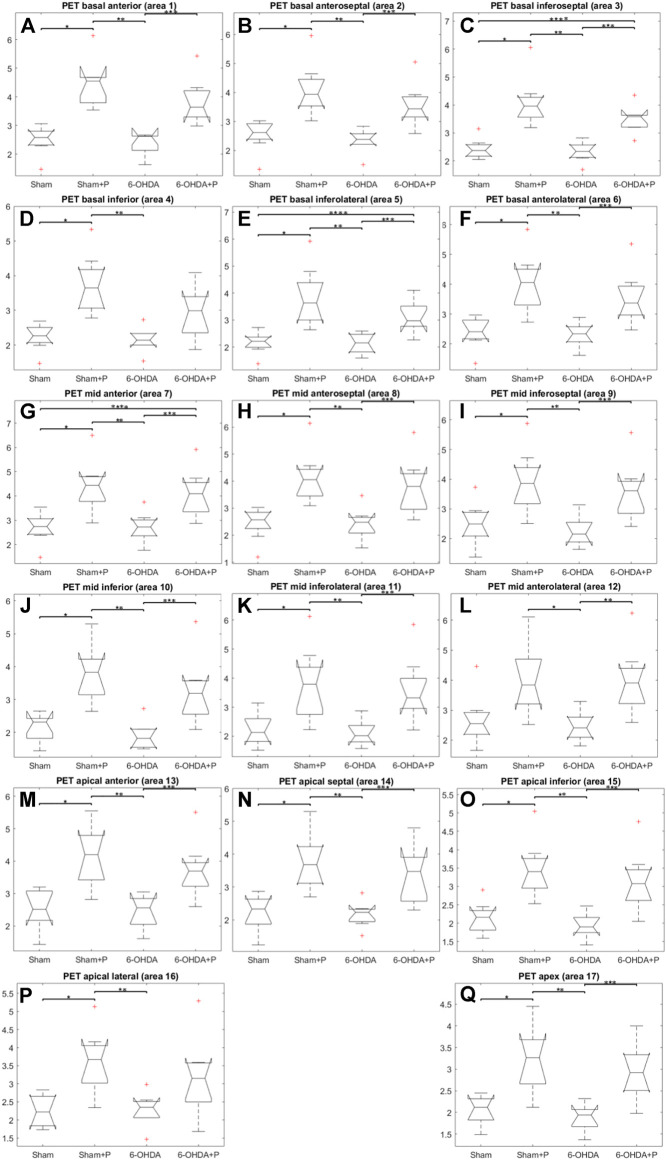
Comparison between the Sham, Sham + P, 6-OHDA and 6-OHDA + P groups of standardized [18F]FDG uptake value. **(A)** *p = 0.003; **p = 0.0014; ***p = 0.028. **(B)** *p = 0.005; **p = 0.0011; ***p = 0.013. **(C)** *p = 0.0011; **p = 0.0014; ***p = 0.04; ****p = 0.037. **(D)** *p = 0.0044; **p = 0.0008. **(E)** *p = 0.003; **p = 0.0014; ***p = 0.011; ****p = 0.021. **(F)** *p = 0.006; **p = 0.002; ***p = 0.013; **(G)** *p = 0.0078; **p = 0.0078; ***p = 0.02; ****p = 0.02. **(H)** *p = 0.006; **p = 0.0029; ***p = 0.014. **(I)** *p = 0.016; **p = 0.0019; ***p = 0.013. **(J)** *p = 0.0074; **p = 0.0011; ***p = 0.012. **(K)** *p = 0.012; **p = 0.0043; ***p = 0.0084; **(L)** *p = 0.01; **p = 0.0089. **(M)** *p = 0.009; **p = 0.0048; ***p = 0.014. **(N)** *p = 0.014; **p = 0.0027; ***p = 0.018. **(O)** *p = 0.007; **p = 0.0009; ***p = 0.006. **(P)** *p = 0.006; **p = 0.0014. **(Q)** *p = 0.01; **p = 0.0008; ***p = 0.007.

At present, Parkinson’s disease (PD) is incurable and available pharmacotherapy has not been proven to retard or end disease progression and its use is frequently hampered by side effects. Many lines of evidence suggest mitochondrial dysfunction, inflammation, oxidative stress, as players of PD pathogenic cascade, that being so, propolis can exert its effects through the synergistic action of a wide range of its chemical compounds (Figure 9) with a variety of pharmacological and biological properties that can act on different target sites related to the pathophysiological processes underlying the disease. In this study, we found that propolis attenuated both the death of dopaminergic neurons in the substantia nigra pars compacta and the loss of striatal fibers in parkinsonian rats. Using PET imaging, we showed that propolis treatment improved glucose metabolism in the heart of rats that consumed it (Figure 10). We also encountered that four metabolites (2-Hydroxybutyric acid, 3-Hydroxybutyric acid, MG (0:0/18:1 (9Z)/0:0 and alanine) were found to be different between groups (6-OHDA, 6-OHDA + P and Sham); and propolis-induced changes in the level of these cardiac metabolites suggest beneficial effects of this dietary supplement.

In this work, bilateral injections of 6-OHDA caused a progressive degeneration of about 58% of the dopaminergic neurons in the substantia nigra and 44% of the striatal fibers, mimicking early human PD. On the other hand, the consumption of propolis minimized neuronal degeneration in the brains of the parkinsonian rats (6-OHDA + P), resulting in the death of 38% of dopaminergic neurons and in the loss of 19% of the striatal fibers. The composition of propolis includes phenolic acid and flavonoids known for their great potential in scavenging free radicals and reactive oxygen species and also for their potent anti-inflammatory action, promoting the preservation of brain tissue against various forms of neurotoxicity (Kureck-Górecka et al., 2013; Bazmandegan et al., 2017; Zhang et al., 2019; Ali and Kunugi, 2020). Highlighted are the caffeic acid phenethyl ester, chrysin and pinocembrine, as they can cross the blood-brain barrier and exert neuroprotective, anti-inflammatory and oxidant effects (Noelker et al., 2005; Shen et al., 2019; Grabska-Kobylecka et al., 2020; Qiu et al., 2020). CAPE is not present in the Brazilian green propolis composition (Hori et al., 2013). On the other hand, caffeic acid, caffeoylquinic acid derivatives and prenylated compounds as artepellin C are substances commonly found in green propolis, that have been reported as potent antioxidants with robust neuroprotective activities (Nakajima et al., 2007, 2009). While the phenolic compounds as caffeic acid ad caffeoylquinic derivatives confer a strong antioxidant activity as a water-soluble profile, artepellin C confers protection against lipid oxidation, as demonstrated in membrane model (Pazin et al., 2020). The current findings reinforce previous data from our research group that revealed a significant reduction in the loss of dopaminergic neurons and fibers from parkinsonian rats by the use of green propolis (Gonçalves et al., 2020).

The understanding of PD needs to be comprehensive, not just derived from the degeneration of the nigrostriatal system, as the non-motor manifestations are intrinsic to the disease encompassing and encompass several organ systems, with autonomic nervous system dysfunction of particular interest (Goldstein, 2014; Cuenca-Bermejo et al., 2021). Cardiovascular changes are a non-motor symptom derived from autonomic dysfunction and commonly seen in PD patients. For example, the reduction in both heart frequency and heart rate variability in humans is well described in the literature (Heimrich et al., 2021; Li et al., 2021; Longinetti et al., 2021) and studies using animal models of 6-OHDA also have mimicked these findings, in addition to bringing other cardiovascular complications as described in human PD (Ariza et al., 2015; Rodrigues et al., 2019; Cabral et al., 2020). In a previous study, our research group found that the use of propolis for 40 days after striatal 6-OHDA lesion provided cardioprotection to PD rats, avoiding a marked decrease in heart rate and its variability (Gonçalves et al., 2020).

Evidence from PD patient cohorts have shown that measurements of brain glucose metabolism using 18F-FDG PET can detect different regional hypometabolism and hypermetabolism pinpoiting disease-related metabolic brain patterns. Therefore, [18F] FDG PET image-based classifications may aid in differentiating idiopathic PD from atypical Parkinsonian disorders, improving clinical practice (Juh et al., 2004; Thobois et al., 2018). A preclinical study using [18F]FDG PET in the unilateral 6-OHDA model of PD found altered cerebral metabolic rate of glucose in basal ganglia, olfactory bulb and amygdala, which are disease relevant brain regions (Silva et al., 2013). To the best of our knowledge, there are no studies that have evaluated cardiac glucose metabolism in PD patients or in animal models of the disease. In the present study, [18F] FDG PET imaging detected higher glucose metabolism in all 17 areas of the left ventricle of rats that consumed propolis in comparison to those rats that did not consume it. Increased metabolism in all propolis-treated rats suggests an effect of dietary intervention in cardiac function. Research indicated that propolis affects metabolic factors and its effects on glycolytic metabolism have been described (Valença et al., 2013; Pahlavani et al., 2020). It has been shown that Brazilian propolis impacts and promotes translocation of insulin-sensitive glucose transporter (GLUT) 4 (Ueda et al., 2013). The transport of glucose in the heart is eminently under the control of GLUT4 (Renguet et al., 2018). Cardiac [18F] FDG uptake is reduced with fasting but rats were not deprived of food in our study. Furthermore, the blood glucose of the animals was measured before PET experiments and glucose levels were not statistically different between the groups of animals.

To provide valuable context when assessing the impact of nutritional interventions, untargeted metabolomics allows to scan metabolites in biological samples, such as heart tissue, and uses statistical metrics to reveal differences of specific compounds and diverse metabolic pathways that reflect metabolic responses to dietary supplements (Mallet et al., 2021). In this study, the decrease in 2-hydroxybutyric acid metabolite levels occurred in the 6-OHDA group, whereas the 6-OHDA + P group exhibited similar metabolite levels to the Sham group. This metabolite is generated by amino acid catabolism and glutathione anabolism, or as a result of the conversion of cystathionine to cysteine (Gall et al., 2010; Ahlin et al., 2021). In situations of greater oxidative stress, cysteine levels increase in the production of glutathione, an important antioxidant that can prevent cell damage caused by reactive oxygen species and free radicals, removing toxic molecules and preventing oxidative damage (Gall et al., 2010; Ahlin et al., 2021). Scenarios associated with enhanced 2-hydroxybutyric acid release may be associated with increased liver glutathione synthesis from methionine as necessary for detoxification functions, suggesting the beneficial cardiac effects of propolis in parkinsonian rats treated with this bee-derived product. Research has shown that propolis protects cell plasma membrane and attenuates the effects of oxidative stress (Nascimento et al., 2018; Laaroussi et al., 2021; Nna et al., 2021). The animals in the 6-OHDA group showed a decrease in the concentration of 2-hydroxybutyric acid in the heart and studies performed in autopsies of humans with PD and in animal models of the disease described a decrease in glutathione content and/or activity as a common finding in PD (Aoyama and Nakaki, 2012; Mazzetti et al., 2015; Dwivedi et al., 2020; Aoyama et al., 2021).

The 3-hydroxybutyric acid metabolite showed no differences regarding its level in the Sham and 6-OHDA groups, however, the 6-OHDA + P group had an increased concentration of this metabolite in the heart. This metabolite is part of the ketone bodies and intermediates the catabolism of amino acids and fatty acids. The anti-inflammatory, energetic and antioxidant effects of 3-hydroxybutyric acid prevent neuronal apoptosis, correct defects in mitochondrial energy production, decrease reactive oxygen species, decrease nitrite content and increase glutathione levels (Majrashi et al., 2021). It has been shown that 3-hydroxybutyric acid protects mesencephalic neurons against the toxic effects of 1-methyl-4-phenylpyridinium, an analogue of heroin that experimentally induces PD (Kashiwaya et al., 2000). The cardioprotective effects of hyperketonemia have been described and, in light of previous findings, 3-hydroxybutyric as important ketone bodies improves cardiac metabolic efficiency, suggesting potential therapeutic actions for these metabolites in the course of impaired cardiac performance (Wei at al., 2022). In this sense, it was previously reported that unified PD rating scale scores were improved by diet-induced hyperketogenic in PD patients (Vanitallie et al., 2005). As PD progresses, 3-hydroxybutyric acid concentration tends to decrease in the patient’s body, suggestive of mitochondrial impairment, pointing to the fact that lifestyle interventions, such as nutrition intervention, that stimulate the production of this metabolite might help to counteract damaging effects associated with disease (Fu et al., 2015; Nielsen et al., 2019; Norwitz et al., 2019; Gonzales-riano et al., 2021). In our study, the beneficial effects of diet-supplementation with propolis are suggested since 3-hydroxybutyric acid cardiac levels were found significantly elevated only in the supplemented animals (6-OHDA + P). Literature shows that the flavonoid chrysin, present in propolis, decreased the rates of low-density lipoproteins, reducing the synthesis of bad cholesterol and helping to regulate enzymes involved in the synthesis of ketone bodies, such as 3-hydroxybutyric acid (Puthanveetil et al., 2021). Besides chrysin, caffeic acid and its derivatives, present in the propolis extract used, displayed actions on cardiovascular system, with marked anti-atherosclerotic and anti-angiogenic effects (Silva et al., 2021).

The Monoacylglycerols (MG) are crucial end-products of dietary triacylglycerol (or triglycerides) digestion. Triacylglycerol is hydrolyzed, culminating in the formation of free fatty acids and MG. Nevertheless, during lipid absorption process, the MG pathway is a major route for the triglyceride formation, since MG are effectively transformed into triglycerides. Lower concentrations of MG (0:0/18:1 (9Z)/0:0) metabolite were found in the 6-OHDA + P group when compared to both the 6-OHDA group and the Sham group. Literature clearly indicates a correlation between plasma triglyceride levels and the risk of coronary heart disease (Do et al., 2013; Fu et el., 2019). Epidemiological and genetic studies in human subjects show that a high concentration of triglycerides is a risk factor for cardiovascular diseases, in light of the fact that the accumulation of triglyceride-rich lipoproteins is causally associated with atherosclerosis and all-cause mortality (Laufs et al., 2019; Sanderasa et al., 2019; Farnier et al., 2021). The distinct compositions and kinds of food impact the amount and release rate of dietary lipids. It is worth noting that in the present study all animals received a fixed chow for laboratory rats composed of corn, nuts, soybean, sunflower seeds, wheat, fortified with vitamins and vegetable oils. Triacylglycerols are important storage lipids in seeds of plants. High concentrations of dietary triacylglycerols may cause an accumulation of MG, as a consequence, the animal diet could explain the high levels of MG found in the rats of the Sham group in our study. On the other hand, propolis exerted a remarkable effect on MG levels in tissues of 6-OHDA + P rats, which differ substantially from those levels found in both 6-OHDA and Sham groups. The study by Oršolić and others showed that 30-day oral supplementation of propolis reduced LDL cholesterol and triglyceride levels in the blood of high-fat diet mice, suggesting a role for propolis in the regulation of triglycerides and cholesterol in these animals (Oršolić et al., 2019).

The alanine metabolite is in lower concentrations in the 6-OHDA group when compared to the 6-OHDA + P and Sham groups, which had similar levels of the metabolite. Alanine is an amino acid, present in the constitution of honey, important for energy metabolism. The increase in alanine concentration in the blood of PD patients indicates a reduction in the rate of gluconeogenesis, preventing an inadequate supply of glucose that can cause neurodegeneration (Dunn et al., 2014; Kori et al., 2016; Cornara et al., 2017). Metabolic pathways involving alanine are associated with glucose consumption, the formation of glutamate and its conversion to glutamine which, if not properly regulated, can lead to excessive oxidative stress and impairment in mitochondrial respiration, energy metabolism and absorption of proteins (Sibson, 2002; Cruzat et al., 2018; Kumari et al., 2020). Therefore, alterations in alanine metabolism, both at high and low concentrations, point to mitochondrial dysfunction in PD (Clarke et al., 2013; Havelund et al., 2017). In addition, mitochondrial dysfunction may depend on external factors such as environmental and also be influenced by hormones controlling glucose metabolism (Moon and Paek, 2015). Perhaps the reported alterations in the alanine-glucose cycle can be evidenced due to the crosstalking between the endocrine and metabolic systems. Cortisol promotes gluconeogenesis by stimulating the synthesis of the key enzyme phosphoenolpyruvate carboxykinase, an opposite effect to that of insulin. For this, it also contributes with the supply of amino acids through the degradation of muscle protein, and with glycerol released from adipose tissue (van Wamelen et al., 2020). Adaptation mechanisms such as the one mentioned have already been reported by Sarabhai and Roden (2019) in stress situations mediated by the crosstalking system as described. The results that describe the alanine metabolite in cardiac tissue may be derived from this interaction between biochemical processes.

From the four cardiac tissue metabolites whose levels were significantly modified between the experimental groups, with the objective of identifying selective molecular signatures and giving meaning to the altered metabolites in relation to the existing biological knowledge gathered in metabolic pathways, we used the MetaboAnalyst web, especially the functional analysis Metabolite Set Enrichment Analysis module to discriminate the relevant metabolic pathways in the present study. Briefly, the propanoate metabolism pathway is related to energy generation and inflammation, mainly in the intestine, contributing to the pathogenesis of PD and can be regulated by a healthy microbiota (Shao et al., 2015; Zhuo et al., 2022). Propanoate metabolism portrays the propionic acid metabolism and is a key carbohydrate metabolism related metabolic pathway. Alanine is a critical glucogenic amino acid involved in sugar and acid metabolism. The glucose-alanine cycle and alanine metabolism pathways actively regulate energy metabolism (Sarabhai and Roden, 2019; Kumari et al., 2020). The glutathione metabolism pathway plays crucial roles in redox signaling and antioxidant defense, cellular homeostasis, regulation of apoptosis, and glutathione depletion is commonly described in neurodegenerative diseases (Smeyne and Smeyne, 2013; Aoyama, 2021; Bjørklundet al., 2021). The urea cycle pathway is related to the elimination of excess nitrogen, as its accumulation impairs the regulation of pro-inflammatory molecules and reactive oxygen species, playing an important role in the onset of neuroinflammatory diseases (Roy et al., 2012; Matsumoto et al., 2019).

In conclusion, Brazilian standardized green propolis (EPP-AF^®^, Apis Flora) increased cardiac glucose metabolism in all propolis-fed rats. This study also identified the cardiac metabolites significantly affected by propolis treatment in parkinsonian rats and provided the relevant metabolic pathways related to the underlying alterations in metabolite abundances. Our pioneering findings bring new insights into the effects of propolis on cardiac metabolism in experimental PD and suggest potential targets for further studies. Treatment with green propolis as a dietary supplement is suggested as a potential candidate to minimize non-motor manifestations of PD.

## Data Availability

The original contributions presented in the study are included in the article/Supplementary Material, further inquiries can be directed to the corresponding authors.
